# A Rare Case of Insulin-Like Growth Factor (IGF-2) Induced Hypoglycemia Associated With Metastatic Colon Cancer

**DOI:** 10.7759/cureus.60211

**Published:** 2024-05-13

**Authors:** Anwar Alshaakh Mohd Mari, Ashlee Sidhu, Moises Matos, Mustafa Kinaan

**Affiliations:** 1 Internal Medicine, HCA University of Central Florida (UCF), Florida, USA; 2 Endocrinology, Diabetes, and Metabolism, University of Central Florida/HCA Healthcare/Orlando VA Medical Center, Orlando, USA; 3 Endocrinology, HCA Florida Osceola Hospital, Kissimmee, USA

**Keywords:** igf-2-induced hypoglycemia, non-insulin-related hypoglycemia, metastatic colon cancer, persistent hypoglycemia, nonislet cell tumor hypoglycemia

## Abstract

The occurrence of hypoglycemia in patients without diabetes is rare, and non-islet cell tumor hypoglycemia (NICTH) accounts for a small portion of these instances. One of the infrequent causes is associated with tumor cell production of Insulin-like growth factor (IGF)-2.

Here is a case of a 66-year-old man with stage IV colon cancer who presented to the emergency department with breathlessness during chemotherapy (Bevacizumab plus FOLFOX4 regimen). He had undergone partial colectomy and chemotherapy three years prior but was recently diagnosed with metastatic liver disease. A CT scan revealed a 15 cm hepatic mass occupying the entire right hepatic lobe. Despite receiving dextrose infusions, he experienced persistent hypoglycemia after meals and during fasting. Given that he had no history of diabetes and denied using any oral hypoglycemic agents, the Endocrinology service was consulted for further evaluation. Plasma blood glucose (BG) was measured at 74 mg/dL (reference range 74-106) during dextrose administration. An 8 AM cortisol test yielded a result of 8.08 mcg/dL (4.30-22.40), ruling out adrenal insufficiency. A 72-hour fast was initiated but terminated at eight hours due to symptomatic hypoglycemia with a plasma BG of 48 mg/dL. C-peptide and Insulin levels were both low, measuring <0.05 ng/mL (0.48-5.05) and <1.0 mU/L (3.0-25), respectively, while beta-hydroxybutyrate (BHB) levels were normal at 1.1 mg/dL (0.2-2.8). Administration of 1 mg glucagon during the fast increased BG to 112 mg/dL within 2 hours. IGF-1 levels were undetectable (<1.9 nmol/L), while IGF-2 levels were at 23 nmol/L (44-129 nmol/L), resulting in an IGF2:IGF1 ratio of 12 (>10), confirming IGF-2 mediated NICTH. Treatment with dexamethasone 10 mg daily was initiated, maintaining blood glucose levels above 70 mg/dL without dextrose infusion.

In approximately 50% of cases of NICTH, the tumor is detected before the onset of hypoglycemia, yet up to half the patients may remain asymptomatic despite having very low BG. Despite having a known hepatic lesion, our patient exhibited minimal symptoms despite severely low BG levels. The mechanisms underlying NICTH may involve tumor secretion of insulin, replacement of hepatic tissue, increased glucose utilization by the tumor, or, most commonly, secretion of IGF-2. In cases of IGF-2-mediated hypoglycemia, insulin, proinsulin, C-peptide, and β-hydroxybutyrate levels are typically low. IGF-2 stimulates the insulin receptors resulting in increased glucose uptake by skeletal muscles and suppression of gluconeogenesis, glycogenolysis, and ketogenesis by the liver. Insulin secretion from pancreatic β-cells is suppressed. IGF-1 levels are usually low, while IGF-2 levels may be high or normal, as many IGF-2omas produce IGF-2 precursors (pro-IGF-2). An elevated IGF-2:IGF-1 ratio (>10) confirms the diagnosis which may be helpful when IGF-2 levels are normal. The primary treatment is through surgical removal or debulking of the tumor. Neoadjuvant therapies such as radiation and chemotherapy may reduce occurrences of hypoglycemia, but only temporarily. Glucocorticoids may be used when the underlying malignancy cannot be treated.

## Introduction

This case was previously presented as a poster presentation at the Endocrine Society’s Annual Meeting ENDO 2023 on June 15, 2023 [1].

The diagnosis of hypoglycemia in patients without diabetes is uncommon. A range of etiologies including adrenal insufficiency, sepsis, liver dysfunction, kidney failure, malnutrition, alcohol-related illnesses, malignancies including islet cell tumors, pancreatic hypertrophy due to bariatric surgery and medications may result in hypoglycemia in non-diabetic and diabetic patients [2]. A careful history and examination can help narrow down the diagnosis. Establishing the presence of Whipple’s triad is an important tool to differentiate between pathologic and physiologic hypoglycemia [3]. It is important to identify and treat the underlying cause of hypoglycemia.

Non-islet cell tumor hypoglycemia (NICTH) represents a minority of these cases, and one of the mechanisms is reported to be tumoral secretion of Insulin-like growth factor (IGF)-2. Upon reviewing the literature, NICTH has been associated with carcinomas including, breast, cervix, lung, gastric, hepatocellular, colon, pancreatic as well as various vascular and mesenchymal tumors [4,5]. IGF-2 is a growth factor sharing structural similarities with insulin and causes similar effects on glucose metabolism. If produced in excess by tumor cells, it can lead to increased glucose uptake by tissues, enhanced glucose utilization, and decreased glucose production by the liver leading to hypoglycemia and symptoms such as confusion, weakness, dizziness, and loss of consciousness [6].

## Case presentation

A 66-year-old man was admitted for evaluation of hypoglycemia following an episode of transient breathlessness, lightheadedness, anxiety, and nausea while receiving chemotherapy. He had no past medical history of diabetes and denied using any oral hypoglycemic agents. He had a past medical history significant for colon cancer, treated with partial colectomy three years earlier. He had been told he was in remission but had failed to have any surveillance follow-up for over a year. Two months earlier, he was hospitalized for new-onset abdominal pain and was found to have metastatic disease to the liver. He re-established care with an oncologist who had initiated treatment with FOLFOX4 plus Bevacizumab. He reported occasional episodes of headaches, fatigue, and anxiety occurring randomly over the past several weeks. He also noticed a loss of appetite and unintentional weight loss for several months.

On admission, vital signs showed BP 152/84 mm Hg, heart rate 93 bpm, respiratory rate of 18 breaths per minute, SPO_2_ 99% on room air, and temperature of 36.7 ºC. His weight was 39 kg with a body mass index (BMI) of 16.25 kg/m^2^ (18.5-24.9 kg/m^2^). On exam, he was in no significant distress but appeared chronically ill and cachectic. Initial labs are listed in Table 1. Further glucose monitoring with fingerstick and venous glucose tests confirmed persistent hypoglycemia with readings as low as 34 mg/dL. Dietary support and dextrose infusion were initiated to maintain euglycemia, and the Endocrinology service was consulted for further evaluation.

**Table 1:** Initial blood work results

Test	Results	Reference Range
Glucose	67 mg/dL	70-100 mg/dL
Aspartate transaminase	138 IU/L	0-40 IU/L
Alanine transaminase	77 IU/L	0-44 IU/L
Alkaline phosphatase	207 IU/L	44-121 IU/L
Albumin	3.3 g/dL	3.4-5 g/dL
Total protein	7.4 g/dL	6.4-8.2 g/dL
Creatine Kinase	96 IU/L	55-170 unit/L
Lactate dehydrogenase	268 IU/L	100-190 unit/L
White blood cell	4.9	4.0-10.5
Hemoglobin	13.2	11.2-15.7
Platelet	367	150-400

Diagnostic assessment

**Figure 1:**
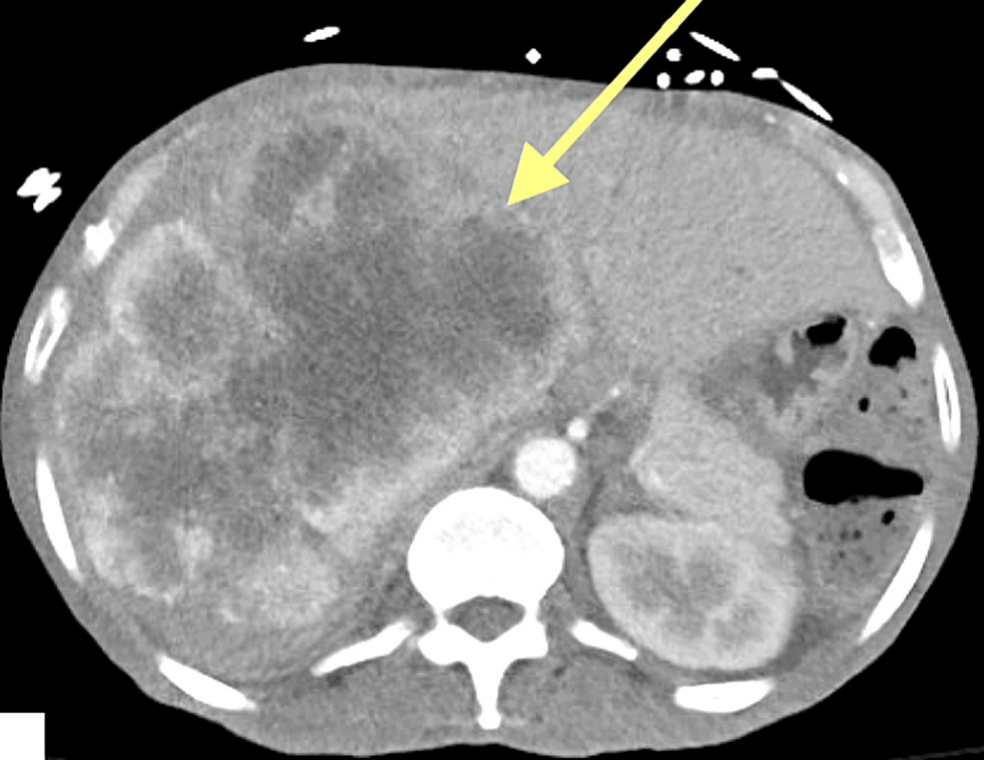
15 cm hepatic mass occupying the entire right hepatic lobe.

CT abdomen with contrast showed a 15 cm hepatic mass occupying the entire right hepatic lobe (Figure 1). Evaluation for adrenal insufficiency and hypothyroidism was unremarkable. Due to the presence of both fasting and postprandial hypoglycemia, a 72-hour fasting test was performed to induce hypoglycemic conditions under medical supervision and to assess whether there is inappropriate overproduction of endogenous insulin. Eight hours into the fast, the plasma blood glucose (BG) dropped to 48 mg/dL and the patient became symptomatic. Labs were drawn, as listed in Table 2. Fasting was then discontinued, and 1 mg glucagon was administered. His BG increased to 112 mg/dL within two hours following glucagon. Table 2 depicts that IGF2 to IGF1 ratio was 12 confirming IGF-2-mediated NICTH.

**Table 2:** Blood work results after eight hours of fasting

Test	Result	Reference range
Glucose	48 mg/dL	70-100 mg/dL
Insulin	<1 mU/L	3.0-25 mU/L
C-peptide	<0.05 ng/mL	0.48-5.05 ng/mL
Beta hydroxybutyrate (BHB)	1.1 mg/dL	0.2-2.8 mg/dL
IGF-1	Undetectable <1.9 nmol/L	7-30 nmol/L
IGF-2	23 nmol/L	44-129 nmol/L

Treatment

Dietary support for cancer-induced cachexia was emphasized. Glucocorticoid treatment with dexamethasone 10mg daily was necessary to maintain euglycemia (>70 mg/dL) off the dextrose infusion.

Outcome and follow-up

The patient was seen in clinic after two months. His BG remained largely well-controlled with dexamethasone, although he did experience some fasting hypoglycemia with morning BG in the 50-60 mg/dL range on several occasions. After further discussion about the prognosis of his disease with his oncologist, the patient elected to forgo further chemotherapy and transition his care to home hospice. To facilitate home BG monitoring, he was started on continuous glucose monitor.

## Discussion

NICTH is a rare condition observed in association with several different solid tumors which can cause severe and persistent hypoglycemia. In approximately 50% of cases, the tumor is identified prior to the onset of hypoglycemia, but up to 50% of these patients may be asymptomatic despite hypoglycemia [7]. Our patient had a known hepatic lesion but was minimally symptomatic at times, despite very low BG. As in patients with insulinoma, hypoglycemia associated with an IGF-2-producing tumor typically presents in the fasting state. IGF-2-linked tumor hypoglycemia has the potential to remain undiagnosed in many cancer patients who are receiving palliative care, where neuroglycopenic symptoms could be incorrectly attributed to the effects of narcotics or other medications [8].

The mechanism of NICTH can include insulin secretion from the tumor, increased glucose utilization by the tumor, replacement of hepatic tissue, or most commonly, tumoral secretion of IGF-2. In cases of IGF-2-mediated hypoglycemia, it is typical for insulin, proinsulin, C-peptide, and β-hydroxybutyrate levels to be low. IGF-2 stimulates the insulin receptors resulting in increased glucose uptake by skeletal muscles and suppression of gluconeogenesis, glycogenolysis, and ketogenesis by the liver. As a result, insulin secretion from pancreatic β-cells is suppressed [8]. IGF-1 levels are usually low, while IGF-2 levels may be high, normal, or low, as many IGF-2omas produce IGF-2 precursors (pro-IGF-2) [8,9]. Assays for pro-IGF-2 are not currently commercially available, however, an elevated IGF-2 to IGF-1 ratio (>10) confirms the diagnosis which may be helpful when IGF-2 levels are normal or low, as in our case [8].

When evaluating non-diabetic patients with hypoglycemia or suspected NICTH, it is still important to rule out causes of endogenous insulin overproduction such as insulinoma. Biochemical evaluation should only be done under confirmed hypoglycemic conditions (serum glucose <50mg/dL), which can be induced through extended fasting or a mixed meal test. The choice between 72-hr fast and mixed meal tests depends on the pattern of hypoglycemia with the former used in patients with predominantly fasting hypoglycemia and the latter used in patients with postprandial hyperglycemia only. In patients with such frequent and severe fasting and postprandial hypoglycemia as our patient, either of these two tests can be performed as long as hypoglycemic conditions are induced to allow testing. We have elected to perform a fast test to achieve hypoglycemic conditions faster, which happened within only eight hours of test initiation. 

The diagnosis of NICTH requires thorough evaluation. It is important to maintain a high index of suspicion in patients with a known malignancy presenting with fasting hypoglycemia, particularly hepatocellular carcinoma which has the highest association with NICTH [8]. Based on our literature review, our case is one of a few published cases of colorectal cancer with liver metastasis associated with NICTH. For early diagnosis and monitoring, the evidence of tumoral overexpression of IGF-2 has prompted an investigation into the utility of IGF-2 measurement in some cancers. IGF2 has also been suggested as a selective marker for colorectal cancer staging and prognosis [10,11]. Treatment primarily involves surgical removal or debulking of the tumor. Temporary reductions in hypoglycemia incidences may be achieved with neoadjuvant treatments like radiation and chemotherapy. However, these effects are fleeting. Glucocorticoids can be used in cases where the underlying malignancy cannot be treated [8]. Glucocorticoids such as dexamethasone help manage the symptoms of hypoglycemia by increasing blood glucose levels. They work by promoting gluconeogenesis in the liver and reducing peripheral glucose uptake. While they do not address the underlying tumor, they can alleviate the symptoms of hypoglycemia and improve the patient's quality of life. Dexamethasone is preferred over prednisone because the latter needs to be metabolized to its active form, prednisolone, in the liver and might be less effective in patients with severe hepatic dysfunction.

## Conclusions

The absence of Whipple’s triad is suggestive of physiologic hypoglycemia where ketogenesis is preserved. The presence of IGFs and Insulin suppress ketogenesis. Fasting hypoglycemia with low C-peptide, insulin and proinsulin and normal ketones is suggestive of NICTH, particularly in the setting of known neoplasia. In patients with persistent hypoglycemia a molar ratio of IGF-2 to IGF-1 of >10 is diagnostic of NICTH.
